# Absence of Rejection in a Facial Allograft Recipient with a Positive Flow Crossmatch 24 Months after Induction with Rabbit Anti-Thymocyte Globulin and Anti-CD20 Monoclonal Antibody

**DOI:** 10.1155/2018/7691072

**Published:** 2018-05-20

**Authors:** Bruce E. Gelb, J. Rodrigo Diaz-Siso, Natalie M. Plana, Adam Jacoby, William J. Rifkin, Kimberly S. Khouri, Daniel J. Ceradini, Eduardo D. Rodriguez

**Affiliations:** ^1^Transplant Institute, New York University Langone Health, New York, NY, USA; ^2^Hansjörg Wyss Department of Plastic Surgery, New York University Langone Health, New York, NY, USA

## Abstract

**Background:**

Donor-specific antibodies (DSA) to human leukocyte antigen increase the risk of accelerated rejection and allograft damage and reduce the likelihood of successful transplantation. Patients with full-thickness facial burns may benefit from facial allotransplantation. However, they are at a high risk of developing DSA due to standard features of their acute care.

**Case Presentation:**

A 41-year-old male with severe disfigurement from facial burns consented to facial allotransplantation in 2014; panel reactive antibody score was 0%. In August of 2015, a suitable donor was found. Complement-dependent cytotoxicity crossmatch was negative; flow cytometry crossmatch was positive to donor B cells. An induction immunosuppression strategy consisting of rabbit antithymocyte globulin, rituximab, tacrolimus, mycophenolate mofetil (MMF), and methylprednisolone taper was designed. Total face, scalp, eyelid, ears, and skeletal subunit allotransplantation was performed without operative, immunological, or infectious complications. Maintenance immunosuppression consists of tacrolimus, MMF, and prednisone. As of posttransplant month 24, the patient has not developed acute rejection or metabolic or infectious complications.

**Conclusions:**

To our knowledge, this is the first report of targeted B cell agents used for induction immunosuppression in skin-containing vascularized composite tissue allotransplantation. A cautious approach is warranted, but early results are promising for reconstructive transplant candidates given the exceptionally high rate of acute rejection episodes, particularly in the first year, in this patient population.

## 1. Introduction

Sensitization with donor-specific antibodies (DSA) to Human Leukocyte Antigen (HLA) has been shown to increase the risk of accelerated solid organ allograft rejection and loss [1]. Several factors may lead to sensitization, including pregnancy, former transplantation, and prior blood transfusions [2]. The latter is of particular relevance to high-surface-area burn patients, who often receive multiple transfusions during acute care. The use of cadaveric skin allografts to provide temporary wound coverage may further increase the likelihood of developing DSA in these patients [3, 4]. While temporary skin allografts may be lifesaving during the acute stage, their immunological effects have made the management of transplant-requiring burn survivors quite challenging.

Vascularized composite tissue allotransplantation (VCA) has become a feasible reconstructive option for patients with severe facial disfigurement. Of the 40 face VCAs performed worldwide, at least eight have occurred in patients that sustained high-surface-area burns involving the central or full face [5]. The number of burn survivors-turned-face recipients is low due to the difficulties that DSA present when listing candidates for transplant, let alone seeking a suitable donor match. At least one burn-survivor face candidate has been withdrawn from the transplant list due to failure to find a donor in 18 months [6]. This has led to the application of desensitization protocols that have proven successful in solid organ transplant experience. Different strategies exist, including but not limited to immunoabsorption, plasmapheresis, intravenous immunoglobulins (IVIG), and monoclonal antibodies [2, 7]. In 2013, a face VCA was performed in a highly sensitized patient who had sustained chemical burns; an induction regimen consisting of rabbit antithymocyte globulin (rATG), bidiurnal total plasma exchange, and IVIG was administered, and the management of a complex antibody-mediated rejection (AMR) episode has been recently described [8].

The most commonly used induction agent to reduce the risk of T cell mediated rejection in high risk patients is rATG [9]. Other T cell targeted induction agents include the monoclonal antibodies daclizumab and alemtuzumab [10]. However, in sensitized patients, the B cell population plays a role in DSA production and AMR development; these phenomena may therefore be prevented using B cell targeted induction agents. A recent prospective trial in sensitized kidney recipients concluded that the addition of (B cell agents) rituximab and/or bortezomib to a rATG-based induction regimen has an acceptable safety profile [11]. In this manuscript, we present the induction immunosuppression and management of a burn-survivor with a positive B cell flow cytometry crossmatch (FCXM), who underwent total face VCA in August of 2015.

## 2. Case Presentation

The patient is a 41-year-old male firefighter who in 2001 sustained a burn injury encompassing the total face, bilateral superior and inferior eyelids, bilateral external ears, lips, and the entire scalp. The patient underwent over 70 autologous reconstructive procedures. Diffuse scar contracture to the periorbital, perioral, and cervical regions resulted in poor facial function, including eyelid apposition for volitional and reflective blink, mastication, phonation, and facial expression. The patient was evaluated by a multidisciplinary team, and voluntarily enrolled in the face transplantation clinical trial at the NYU Langone Medical Center (clinicaltrials.gov number NCT02158793; Institutional Review Board protocol # I14-00550).

On August 12, 2015, the donor family granted permission for a face transplantation evaluation. The donor, an otherwise healthy 26-year-old male who suffered irreversible traumatic brain injury, met preset criteria based on age, sex, general health, skin color match, and craniofacial dimensions. Serology was positive for cytomegalovirus and Epstein-Barr virus for both donor and recipient; recipient panel reactive antibody (PRA) score was 0. The donor was ABO identical to the recipient, and HLA antigen mismatch was 2-2-2 for A, B, and DR (Donor: A3, 30; B13, 47; C6; DR4, 13, 52, 53; Recipient: A1, 11; B8, 44; C5, 7; DR13, 17, 52; and DQ2, 6). Complement-dependent cytotoxicity crossmatch (CDCXM) was negative for T and B cells. FCXM was negative for T cells but repeatedly positive for donor B cells. Median channel displacement (MChD) was 110, with positivity defined as MChD ≥ 50 at our HLA laboratory. Presence of a class II DSA was ruled out with high resolution allele subtyping. Minor antigen HLA and endothelial cross matching was not performed.

**Figure 1:**
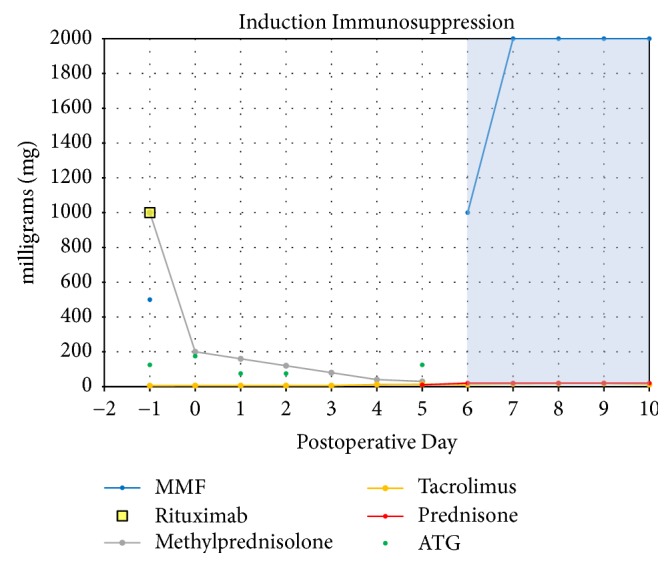
Graphic representation of induction immunosuppression regimen for presented face transplant recipient. The cumulative dose of rabbit antithymocyte globulin (ATG) reached 6 mg/kg (total of 575 mg). Patient also received tacrolimus and mycophenolate mofetil (MMF) en route to the operating room for the facial transplant procedure. The shaded area indicates the initiation of maintenance immunosuppression.

Based on FCXM results, an induction regimen was designed consisting of rATG 6 mg/kg (575 mg total dose), methylprednisolone taper, and the anti-CD20 agent rituximab (1 g) (Figure 1). The patient also received intraoperative tacrolimus (5 mg) and mycophenolate mofetil (MMF) (1000 mg). Total face, scalp, eyelid, ears, and skeletal subunit allotransplantation was performed on August 14, 2015; surgical and technical details have been described [12]. Infection prophylaxis with trimethoprim-sulfamethoxazole (160 mg/day), valganciclovir (450 mg/day), and micafungin (100 mg/day) was administered; piperacillin/tazobactam and clindamycin were also given for oral infection prophylaxis. Maintenance immunosuppression consists of tacrolimus, MMF, and prednisone. Immunosuppression, renal function, and lymphocyte subset counts (CD3+, CD19+) were recorded. For biopsies of allograft skin taken on POD 410 and POD 711, tissue hematoxylin and eosin stains and C4d immunohistochemistry staining were performed by the histology laboratory of the Department of Surgical Pathology at Tisch Hospital of the NYU Langone Medical Center. Rabbit polyclonal C4d anti-human antibody (Cell Margue) was used. The Ultraview Universal DAB Detection Kit (Ventana Medical Systems, Inc.) was used for identification.

In the first 24 months after transplant, the patient has not presented acute rejection episodes (Table 1), infectious, metabolic, or other immunologic complications. Planned secondary surgeries were performed, which have optimized aesthetic and functional improvements (Table 2). After steady state tacrolimus levels were achieved, micafungin was replaced with fluconazole (200 mg/day) on postoperative day (POD) 25.

**Figure 2:**
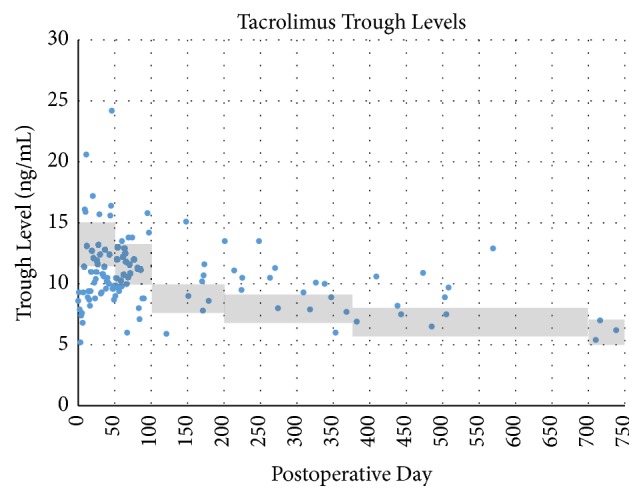
Patient's maintenance tacrolimus trough levels recorded in the posttransplant period. Shaded area represents the target trough levels, which have gradually decreased over time.

**Figure 3:**
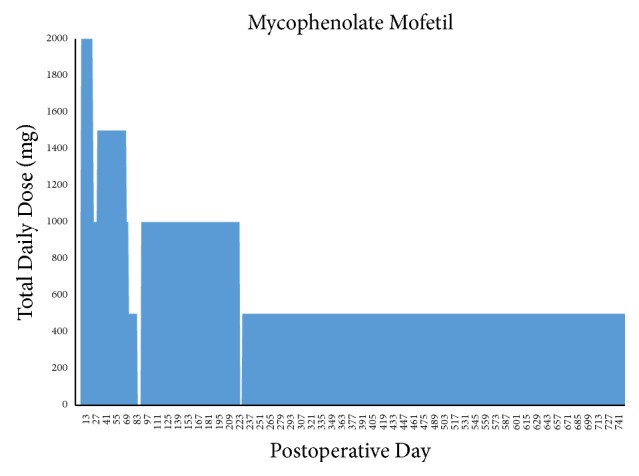
Patient's total maintenance dosage of mycophenolate mofetil. Dose reductions were made to mitigate leukopenia requiring filgrastim treatment. Brief treatment interruptions were made in the setting of suspected viral gastroenteritis (postoperative day 87–91) and suspected viral bronchitis (postoperative day 227–229).

**Figure 4:**
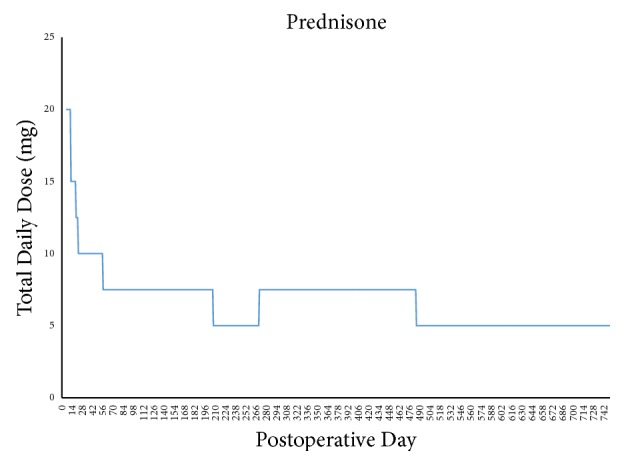
Patient's total maintenance dosage of prednisone during posttransplant period.

Maintenance immunosuppression has remained stable, with progressive decreases in tacrolimus trough target levels (Figure 2) as well as MMF and prednisone doses (Figures 3 and 4). As of postoperative month 24, tacrolimus target levels are 5–7 ng/mL; the patient is receiving, 250 mg BID of MMF and 5 mg/day prednisone.

**Figure 5:**
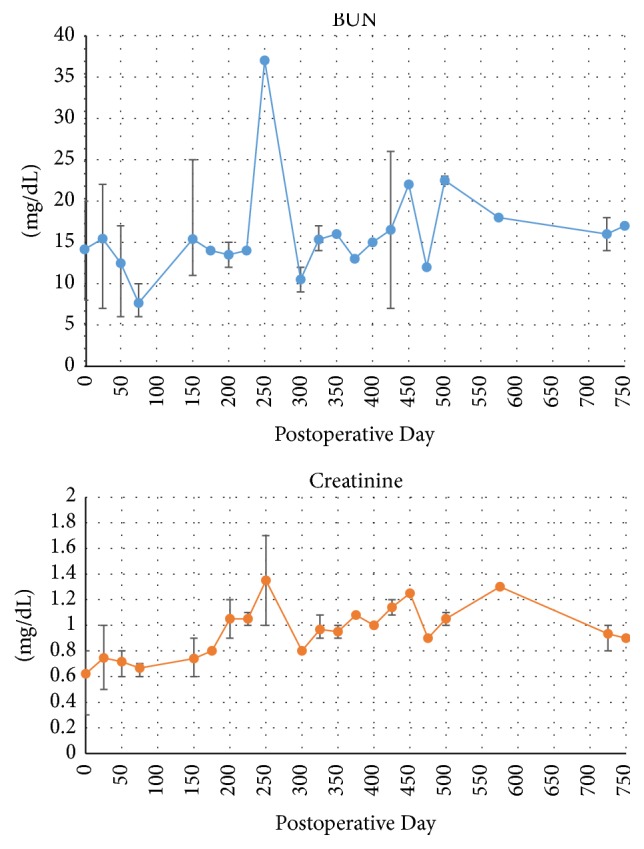
Patient's posttransplant renal function represented by blood urea nitrogen (BUN) and creatinine levels. Bars represent range of values in a 25-day period; plotted points represent the mean value during the same time period.

The patient's renal function has remained stable throughout the first 24 months after transplant (Figure 5).

**Figure 6:**
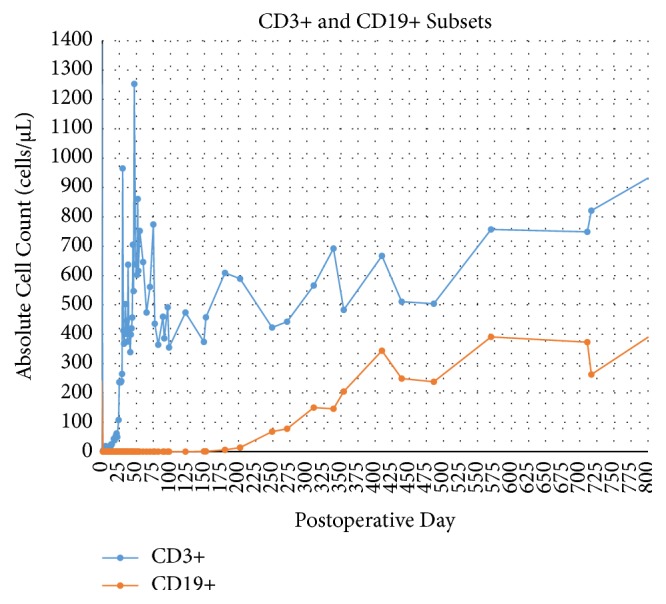
CD3+ and CD19+ lymphocyte subset studies shows depletion of T cells and B cells immediately after induction with rabbit antithymocyte globulin and rituximab. Gradual B cell repopulation began at approximately postoperative day 200, as expected. T cell repopulation is apparent at approximately postoperative day 25; patient has presented prolonged leukopenia.

Lymphocyte subset studies show reestablishment of the T lymphocyte (CD3+) population on approximately POD 25, soon after completion of induction regimen. The B lymphocyte (CD19+) population reappeared in laboratory studies after approximately POD 200 and has undergone gradual reconstitution. The patient has presented with prolonged neutropenia, treated with filgrastim regimen as needed (Figure 6).

**Figure 7:**
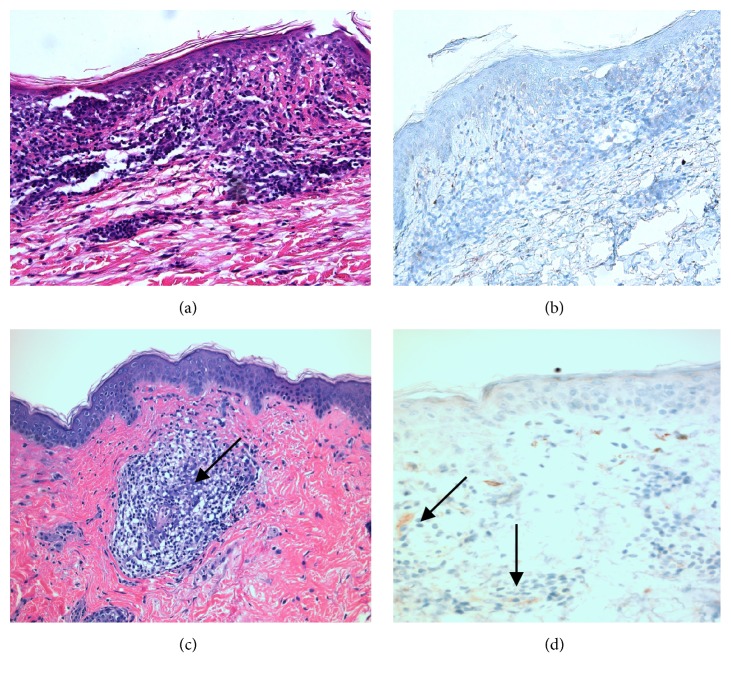
Hematoxylin and eosin (H&E) staining revealed perivascular dermatitis with eosinophils at our 1 year time point (a) and perivascular lymphocytic dermatitis at the 2-year mark (c); C4d staining was negative at our 1 year time point (b) compared with sparse but focal superficial dermal positivity at 2 years after transplant (d). Black arrows indicate inflammatory infiltrates which correspond with focal C4d positivity.

Histopathology analysis of allograft skin biopsy performed on POD 410 (Table 1) showed sparse perivascular dermatitis with eosinophils (Figure 7(a)), consistent with Banff grade 0-1; C4d immunohistochemistry staining was negative (Figure 7(b)). Histopathology of allograft skin biopsy performed on POD 711 (Table 1) showed focal perivascular lymphocytic dermatitis (Figure 7(c)), consistent with Banff grade 0; C4d immunostain showed focal positivity in the superficial dermis (Figure 7(d)).

## 3. Discussion

The most frequent comorbidity in hand and face VCA is acute rejection, and the incidence exceeds 80%, significantly higher than that of abdominal and thoracic organ allografts [13]. The overwhelming majority of face VCA recipients experience an episode of acute rejection in the early posttransplant period, and almost all experience at least one episode of rejection in the first year [14]. Early results in face VCA have demonstrated that most rejection episodes have been successfully medically managed with high-dose steroids and/or increased maintenance immunosuppression, allowing for excellent short and intermediate graft survival in this limited patient cohort [5]. The long-term effect of early rejection episodes on the enduring functional and aesthetic outcome of the graft and patient survival is yet to be determined.

HLA matching and minimizing rejection risk should be the most compelling factor in donor selection. A 2012 study demonstrated a link between the number of acute rejection episodes and HLA mismatches in VCA, though statistical significance was not achieved with the limited sample size [15]. With the exception of uterus transplantation, VCA is unique compared to solid organ transplantation due to the necessary consideration of expected visual, functional, and aesthetic characteristics of the allograft. Characteristics like matching skin tone, age, size, and acquired visual attributes may further restrict an already limited pool of suitable donors; finding an ideal immunologic match is challenging, and ideal HLA matching is generally not feasible. The usual priority for optimal immunologic match, as seen in solid organ transplantation, becomes even more difficult due to the small percentage of donors that consent to VCA donation. Furthermore, virtually all face VCA candidates have had multiple sensitizing events prior to or during the treatment of their original injury, such as blood transfusions, allogeneic skin grafting, or pregnancy. Available options to preemptively lower the risk of rejection, that is, desensitization therapy, are difficult to achieve due to the unpredictable timing of transplantation and limited VCA donor pool.

While the presented recipient was on the transplant waitlist, several willing donors were ruled out due to immunologic unsuitability, as evidenced by positive FCXM and the confirmed presence of DSA. In addition, a potential donor with an excellent immunologic match was disqualified as a result of disparate skin tone and the potential psychological impact of the presence of facial tattoos extending beyond the hairline. Ultimately, the donor selected after more than 12 months on the waitlist was an ideal age and skin tone match but presented a repeatedly positive B cell FCXM. We considered this donor to be within acceptable immunologic risk, given the ideal donor-recipient match based on all other parameters. The facial allograft, which included the entire scalp, ears, and eyelids, was more extensive than all previously reported face VCA, and there was initial concern that this alone could also increase the risk of rejection.

Until recently, a positive crossmatch was considered an absolute contraindication to VCA due to the unknown impact of AMR on skin-containing allografts. However, a face VCA recipient with positive T and B cell FCXM, weakly positive T cell CDCXM, and confirmed presence of DSA underwent induction therapy with thymoglobulin and plasmapheresis. The patient experienced acute rejection on POD#5, initially treated with thymoglobulin; the episode progressed to refractory Banff grade III rejection, ultimately controlled with several months of therapy with plasmapheresis, eculizumab, bortezomib, and alemtuzumab [8]. Extrapolating data from immunologic risk stratification for renal transplantation [16] (Table 3), the selected donor for our face VCA candidate was categorized as moderate risk. After extensive discussions with our patient, we considered a positive FCXM, a negative CDCXM, and absence of DSA acceptable for transplantation, as the donor presented exceptional skin tone, age, size, and gender match.

Donor skin immunogenicity [17] and resident donor T cells in the facial allograft [18] have been characterized as major contributors to the rejection of skin-containing VCAs. Direct antigen presentation by resident donor lymphocytes in the allograft produces a more robust immune response than indirect presentation of donor antigens by recipient lymphocytes [18]. Targeting the B lymphocyte population with induction immunosuppression has not previously been described in VCA. B cells likely play an important role in the immune response to an allograft beyond antibody production, particularly by supporting T cells [19]. Induction regimens in the facial VCA literature have mainly targeted T cell populations alone; the majority of groups have utilized polyclonal ATG or interleukin 2 (IL-2) receptor antagonists (basiliximab), with certain groups also including plasma exchange, IVIG, graft irradiation, or hematopoietic stem cell transplantation [20]. Despite these induction regimens proving successful in solid organ transplantation, early rejection rates remain very high in the VCA cohort.

Our initial plan for induction for a patient with a negative crossmatch was rATG alone. The presence of a positive B cell FCXM implied an increased risk of rejection for a procedure with already unacceptably high rates of early rejection compared to other transplanted organs. Depletion of the recipient's B cell population, including memory B cells, would potentially reduce the risk of rejection in the early posttransplant period, particularly in the presence of a positive B cell FCXM. We hypothesized that targeted depletion of the resident donor B cells within the graft may further reduce the risk of acute rejection by limiting highly immunogenic direct antigen presentation (ref). Rituximab, a chimeric murine/human monoclonal antibody directed against the CD20 pan-B cell marker expressed on pre-B and mature B-lymphocytes, induces lysis of CD20-expressing B cells in peripheral blood as well as tissues. Existing DSA titers are not affected, but de novo alloantibody formation is prevented. Clonal B cell expansion in rejection episodes is also inhibited. The drug is well tolerated by patients; its effects last for over 6 months, and it can be monitored by following CD19/CD20 counts [21].

The majority of VCA recipients have received maintenance triple therapy consisting of a calcineurin inhibitor (CNI), MMF, and steroids [5, 15, 20]. In some patients, CNI has been replaced with mechanistic target of rapamycin (mTOR) inhibitors to avoid nephrotoxicity, though a significantly increased incidence of acute rejection has been observed in hand VCA experience [15]. A primary goal in developing our immunosuppression strategy was to avoid early rejection episodes, particularly in the first 30–60 days. Typical triple drug therapy was planned, utilizing CNI, antimetabolite, and corticosteroid with a prolonged dose deescalation timeline. Goal tacrolimus trough levels between 11 and 15 ng/mL were planned for the first 6 weeks. A gradual decrease in tacrolimus target trough level was followed according to Table 4. Anticipated dose adjustments, planned in the event of medication intolerance or impaired renal function, have not been necessary. A 2000 mg total divided dose of MMF was initiated in the early postoperative period, though dose reductions were necessary due to chronic leukopenia, likely attributed to the bone marrow-suppressive effects of MMF and valganciclovir, requiring regular filgrastim injections (Figure 3). Methylprednisolone was tapered and converted to oral prednisone (10 mg daily) by POD#5. This was further reduced to 7.5 mg daily until all planned operative revisions were completed; the patient is currently maintained on 5 mg/day. The rationale for a prolonged taper of immunosuppression stems from VCA reports of high rates of rejection episodes between the second and third posttransplant month, when the effects of lymphocyte depletion begin to abate [20].

In solid organ transplantation, the diagnosis of AMR requires a combination of clinical exam, histopathology, DSA, and C4d staining [22]. In VCA, however, AMR has been rare [8], and chronic rejection is not well understood. A recent report of AMR in a face VCA recipient describes de novo development of DSA, followed by maculopapular allograft lesions confirmed by histopathology and positive C4d stains [23]. In our patient, positive C4d immunostaining was an isolated finding on a planned allograft biopsy performed on POD 711; no clinical signs of rejection were observed, histopathology was not consistent with acute rejection, and the patient has not developed DSA. While the overall context suggests that the patient has not developed AMR, this isolated finding is of unclear significance. A study of more than 800 liver recipients concluded that a positive C4d staining was an imperfect marker for AMR, requiring correlation with histopathology and clinical presentation; in DSA-negative recipients, this finding probably represents a nonalloantibody insult [24]. In addition, a single center study following over 200 heart transplant recipients found that the presence of an isolated C4d positive stain in myocardial capillaries was not associated with worse long-term outcomes [25]. Nevertheless, further investigation is necessary to determine the role of C4d staining in skin-containing VCA.

## 4. Conclusions

This is the first reported facial VCA utilizing an induction regimen of rATG in combination with rituximab. There have been no acute rejection episodes, opportunistic infections, or renal complications in the first 24 months after transplant. While the efficacy of this regimen cannot be concluded by a single case, these results are promising. Acute rejection in face VCA remains a clinical challenge, with unacceptably high rates reported in early series. Further understanding of VCA-specific risk factors for rejection and improved induction and maintenance immunosuppression regimens is necessary for future success. In addition, our findings highlight the importance of clinical context and exam in the evaluation and diagnosis of acute VCA rejection. Moreover, future investigation is warranted to understand the role of isolated C4d staining in VCA, which remains unclear. While acute rejection episodes have been manageable in global VCA experience, the long-term effects of acute rejection episodes have yet to be fully determined. Prevention of rejection episodes may improve long-term outcomes as has been seen in solid organ transplantation (ref) [26]. We are cautiously optimistic that induction targeting B and T lymphocyte depletion, along with conservative tapering of maintenance immunosuppression, may reduce the rate of early rejection in facial VCA and make its reconstructive benefits available to sensitized patients that were once considered at too high a risk for transplantation.

## Figures and Tables

**Table 1:** Timeline of surgeries performed after facial allotransplantation.

Post-operative day (POD)	Procedure performed
POD 0	Total face, eyelids, ears, scalp, and skeletal subunit allotransplantation

POD 1	Evacuation of Hematoma in the neck

POD 11	Debridement of posterior scalp

POD 39	Debridement and advancement of posterior scalp; Advancement of upper and lower lip; Debridement of nose and eyelids

POD 172	Intraoral tissue debulking; Ectropion release of bilateral lower eyelids and primary repair

POD 241	Brow lift; Tracheostomy reversal; Placement of endosseous dental implants to posterior mandible

POD 318	Submental lipectomy; Uncovering of right mandibular endosseous implants

**Table 2:** Timeline and findings of skin biopsies performed after facial allotransplantation.

Post-operative day (POD)	Histopathological Findings	Banff Grade	Clinical Presentation
POD 3	Sparse perivascular mononuclear cell infiltrate; no rejection	0	Planned biopsy; no clinical signs or symptoms.

POD 95	Superficial perivascular lymphocytic infiltrate; no rejection	0	Mild facial swelling and erythema resolved after administration of diphenhydramine; presumed allergic response.

POD 171	Sparse perivascular lymphocytic infiltrate with rare eosinophils; no rejection	0	Planned biopsy prior to outpatient surgery; no clinical signs or symptoms.

POD 410	Sparse superficial perivascular dermatitis with eosinophils; “early rejection cannot be excluded” but likely drug eruption	0-1	

**Table 3:** Risk of rejection based on crossmatch results and presence of donor-specific antibody.

Rejection Risk	CDC XM	FC XM	DSA
High	+	+/−	+/−
High Moderate	−	+	+
Moderate	−	+	−
Low Moderate	−	−	+
Low	−	−	−

CDC XM, complement dependent cytotoxicity crossmatch; FCXM, flow cytometry crossmatch; DSA, donor specific antibody; +, positive/present; −, negative/absent. Presence of DSA defined as mean fluorescence intensity (MFI) > 2000.

**Table 4:** Target tacrolimus trough levels.

Post-operative day	Tacrolimus trough target level (ng/mL)
1–45	11–15
46–100	10–13
101–200	8–10
201–365	7–9
365 and beyond	6–8

## Abbreviations

AMR: Antibody-mediated rejection
BID: * Bis in die*
CNI: Calcineurin inhibitor
CDCXM: Complement-dependent cytotoxicity crossmatch
DSA: Donor-specific antibodies
FCXM: Flow cytometry crossmatch
HLA: Human Leukocyte Antigen
IL-2: Interleukin 2
IVIG: Intravenous immunoglobulins
mTOR: Mechanistic target of rapamycin
MChD: Median channel displacement
MMF: Mycophenolate mofetil
PRA: Panel reactive antibody
POD: Postoperative day
rATG: Rabbit antithymocyte globulin
VCA: Vascularized composite tissue allotransplantation.
